# Exercise Ameliorates Renal Cell Apoptosis in Chronic Kidney Disease by Intervening in the Intrinsic and the Extrinsic Apoptotic Pathways in a Rat Model

**DOI:** 10.1155/2013/368450

**Published:** 2013-09-09

**Authors:** Kuan-Chou Chen, Chiung-Chi Peng, Chiu-Lan Hsieh, Robert Y. Peng

**Affiliations:** ^1^Department of Urology, School of Medicine, College of Medicine, Taipei Medical University, 250 Wu-Shing Street, Taipei 11031, Taiwan; ^2^Department of Urology, Taipei Medical University-Shuang Ho Hospital, Taipei Medical University, 291 Zhongzheng Road, Zhonghe, Taipei 23561, Taiwan; ^3^Graduate Institute of Clinical Medicine, College of Medicine, Taipei Medical University, 250 Wu-Shing Street, Taipei 11031, Taiwan; ^4^Graduate Institute of Biotechnology, Changhua University of Education, 1 Jin-De Road, Changhua 50007, Taiwan; ^5^Research Institute of Biotechnology, Hungkuang University, 34 Chung-Chie Road, New Taichung City, Taiwan

## Abstract

We hypothesized that doxorubicin (DR) induced chronic kidney disease (CKD) could trigger the intrinsic and the extrinsic renal cell apoptotic pathways, while treadmill exercise could help prevent adverse effects. Male Sprague-Dawley rats were subjected to treadmill running exercise at a speed of 30 m/min, 30 or 60 min/day, 3 times per week, for a total period of 11 weeks. The physiological and biochemical parameters were seen substantially improved (DR-CKD control, 30 min, 60 min exercise): the ratio of kidney weight/body weight (0.89, 0.74, and 0.72); the WBC (1.35, 1.08, and 1.42 × 10^4^ cells/**μ**L); RBC (5.30, 6.38, and 6.26 × 10^6^ cells/**μ**L); the platelet count (15.1, 12.8, and 11.3 × 10^5^/**μ**L); serum cholesterol (659, 360, and 75 mg/dL); serum triglyceride (542, 263, and 211 mg/dL); BUN (37, 25, and 22 mg/dL). Bcl-2 and intramitochondrial cytochrome *c* were upregulated, while the levels of Bax, SOD, MDA, cleaved caspases 9, 3, 8, 12, and calpain were all downregulated in DRCKD groups with exercise. CHOP (GADD153) and GRP78 were totally unaffected. FAS (CD95) was only slightly suppressed in the 60 min exercise DRCKD group. Conclusively, exercise can ameliorate CKD through the regulation of the intrinsic and extrinsic apoptosis pathways. The 60 min exercise yields more beneficial effect than the 30 min counterpart.

## 1. Introduction

Estimates of the global burden of disease indicate that diseases of the kidney and urinary tract account for approximately 830,000 deaths and 18,467,000 disability-adjusted life years annually, ranking them the 12th among causes of death (1.4 percent of all deaths) and the 17th among causes of disability (1.0 percent of all disability-adjusted life years) [1]. Chronic kidney disease (CKD) patients often suffer from cardiovascular or cerebrovascular disease, and their deaths may be attributed to either complication [2]. 

Different types of CKD usually undergo a progressive process to develop glomerular sclerosis and/or renal interstitial fibrosis, finally inducing kidney failure [3]. Generally, renal diseases will progress to a final stage generally called the end-stage renal disease, and the remaining function has to be substituted by renal replacement therapy.

There is a speculation that in kidney disease there are many signaling biomarkers related to cell death through proapoptotic processes; that is, the processes are associated with the intrinsic and the extrinsic pathways. The intrinsic pathway involves both the endoplasmic reticulum (ER) and mitochondrial pathways. The ER pathway actually involves Bip-GRP78, caspase-12, caspase-9, and caspase-3, while the mitochondrial pathway involves mainly Bcl-2, Bax, cytochrome *c*, caspase-9, and caspase-3. Alternatively, the extrinsic pathway begins with the activation of Fas-CD95, and from caspase-8 to caspase-3 [4]. Otherwise, the cell survival pathway involves PI3 K, Akt, and p-caspase-9 [5].

Interventions to ameliorate or reverse a decline in renal function prior to reaching end-stage renal failure could potentially reduce the incidence of renal failure. Accumulating evidence has suggested that exercise can attenuate and/or reverse the proapoptotic process in the kidney [6]. Specifically, moderate intensity exercise has been shown to provide cardiovascular and metabolic benefits and to increase in the endurance time of predialysis patients [7–10]. In contrast to this, other studies have indicated that exercise does not alter age-dependent renal disease [11]. The reasons for this discrepancy may be related to the strenuousness of the exercise [6]. 

Doxorubicin (DR, commercial name Adriamycin) has been used as an anticancer (antineoplastic) medication. It interferes with cancer cell growth and slows their migration in body [12]. DR had been used to induce nephropathy as a model of chronic progressive glomerular disease, [13] generally named “the chronic kidney disease (CKD).”

We hypothesize that exercise could attenuate or reverse the signaling processes in CKD which may involve both the intrinsic and extrinsic apoptotic pathways. To test this hypothesis, doxorubicin (DR) was used to induce a CKD model in rats [13]. The proapoptotic and prosurvival proteins involved in the two pathways were examined.

## 2. Methods

### 2.1. Chemicals and Kits

All biochemical tests were conducted using enzymatic colorimetric assays with specific kits provided by Roche (Basel, Switzerland). Doxorubicin (Adriamycin) was a product of Pfizer (Milano, Italy). Pro-PREP lysis buffer was purchased from iNtRON Biotechnology (Seongnam, Korea). The kit source for other determinations included superoxide dismutase (SOD) and thiobarbituric acid reactive substance (TBAR) from Cayman (Ann Arbor, MI, USA). Rat antibodies for caspase-3, BcL-2 (1 : 1000), BAX (1 : 1000), glyceraldehyde 3-phosphate dehydrogenase (GAPDH) were purchased from Cell Signaling (Danvers, MA, USA). *β*-actin was provided from Novus Biologicals (Littleton, CO, USA). Cytochrome *c* was supplied by Biovision (Milpitas, CA, USA). Caspase-8 and caspase-9 were products of Santa Cruz (Santa Cruz, CA, USA). Caspase 12 was produced by Abcam (Cambridge, UK). CD95 was provided by Millipore (Billerica, MA, USA); and *μ*-calpain (Calpain 1 (small subunit): CAPNS1 antibody), 78 kDa glucose-regulated protein (GRP 78), and C/EBP homologous protein (CHOP) were manufactured by Epitomics (Burlingame, CA, USA). Chemiluminescent horseradish peroxidase (HRP) substrate was the product of Millipore (Billerica, MA, USA). Cobalt chloride stock solution (25 mM cobalt chloride in distilled water), TdT reaction buffer (25 mM Tris-HCl, 200 mM sodium cacodylate, 0.25 mg/mL BSA, and 1 mM cobalt chloride), enzyme reagent, label reagent, TdT reaction mixture, and stop wash buffer (300 mM NaCl, 30 mM sodium citrate) were all supplied by Roche Diagnostic (Indianapolis, IN, USA).

### 2.2. Animal DRCKD Model and Treadmill Exercise Training Protocol

The experimental protocol was approved by the China Medical University Ethics Committee of Experimental Animals (Taichung, Taiwan). Principles of laboratory animal care (NIH publication) were followed. Thirty-six four-week-old Sprague-Dawley male rats (BioLASCO Co., Ltd., Taipei, Taiwan) weighing 225–250 g were used in the study. The rats were acclimated and fed ordinary laboratory chow during the first week. The rats were housed in the animal room and maintained at a relative humidity of 60–75% within 23 ± 1°C with a 12 h/12 h light/dark cycle. The animals were allowed free access to water and ordinary laboratory pellet chow containing 1.8–2.2% calcium, 1.15% phosphorus, and 2650 kcal/kg energy. The rats were randomly assigned to six groups of 6 rats each as follows: group 1, sedentary; group 2, 30 min exercise; group 3, 60 min exercise; group 4, sedentary with doxorubicin-induced CKD (DRCKD); group 5, DRCKD plus 30 min exercise; and group 6, DRCKD plus 60 min exercise. These six groups were separately housed in twelve colony cages, with 3 rats in each. The acclimation to exercise was started from the second week, starting from 10 min, 20 min, 30 min to 50 min per time three times per week. CKD was induced by a single subcutaneous injection of 8.5 mg/kg of doxorubicin during the third week. This has been confirmed to be the optimum dose of DR to induce CKD in SD rats [13]. The regular exercise protocol was continued thereafter for 30 min for groups 2 and 5 and 60 min for groups 3 and 6. The sedentary groups (groups 1 and 4) remained in cages under the same environmental conditions and were inspected daily. DR induces thinning of the glomerular endothelium and podocyte effacement associated with loss of size- and charge-specific barriers to filtration of plasma proteins. These changes are seen as early as 1-2 weeks after DR injection and are severe by 4 weeks [14]. Our experiment was performed for a total period of 13 weeks according to Okuda et al. with a slight modification [13]. Briefly, week 1 was used for acclimation and week 2 for exercise acclimation. DR was administered during week 3. Exercise therapy was started immediately after DR administration; so the total period covered a span of 11 weeks (from week 3 to week 13) [13].

### 2.3. Blood Collection and ELISA of SOD and TBARs

Blood samples were withdrawn from the abdominal aorta under ether anesthesia. The blood sample was centrifuged at 3000 ×g to separate the serum. The sera obtained were used to perform cell counting for WBC, RBC, and platelet. The common biochemical measurements were also conducted for cholesterol, triglyceride, and BUN using an automatic analyzer (Ciba-Corning Express Plus) (Ciba-Corning, USA) and reagent (Siemens, Bakersfield, CA, USA). ELISA was used for determination of SOD and malondialdehyde. All ELISA protocols were performed following the manufacturer's instructions. A SYSMEX K-1000 reader (San-Tong Instrument Co., Taipei, Taiwan) was used. 

### 2.4. Tissue Collection

After euthanization, the organs were visually inspected for outer morphological changes. The kidneys were excised, rinsed twice with saline, with the adhering water sucked with soft tissues, and weighed. The kidneys obtained were immediately frozen with liquid nitrogen and stored at −80°C. 

### 2.5. Cleaved Caspases-3, -8, and -9 Immunohistochemical Examinations

Kidneys were fixed by immersion with 10% formalin in phosphate-buffered saline (PBS) (pH 7.4) at 4°C for 24 h and processed for paraffin embedding. Paraffin-embedded sections 3-*μ*m thick were deparaffinized in xylene, rehydrated in graded ethanol, and washed in 0.1 mol/L PBS (pH 7.5). The sections were then incubated with 3% H_2_O_2_ for 10 min at room temperature and washed three times with distilled water. Antigen retrieval was performed by heating the sections twice in 200 mL antigen retrieval citrate buffer in a microwave oven at 98°C for 8 min. After cooling, the sections were blocked with 1% normal goat serum in PBS for 20 min.

The slides were then incubated with primary antibodies against cleaved caspase-3, -8, and -9 at 4°C for 16 h, and the sections were washed in PBS and incubated with a HRP polymer anti-mouse/rabbit antibody (Novus Biologicals, Littleton, CO, USA) for 20 min at room temperature. After incubation, the sections were rinsed in PBS and developed by 0.04% 3, 3-diaminobenzidine tetrachloride (Sigma-Aldrich Co., St. Louis, MO, USA). Finally, all sections were counterstained with hematoxylin and visualized using an Olympus light microscope (Tokyo, Japan). Quantitative analysis was performed using an Image-Pro-PLUS (Media Cybernetics, Inc., Rockville, MD, USA) analysis system at ×400 magnification. Thirty glomeruli in the cortex and the cortex-medulla junctions were randomly scanned. The integrated optical absorbance (IOA) was measured. The sum of the IOA was obtained and the mean value was analyzed. 

### 2.6. Western Blotting

To prepare proteins for immunoblot analyses, frozen renal cortex tissue samples (approximately 100 mg) were homogenized with homogenizer (T10 basic, IKA Co., Staufen, Germany) in 1 mL of Pro-PREP lysis buffer (50 mM Tris, pH 7.2, 150 mM NaCl, 1 mM EGTA, 5 mM EDTA, 0.5% Triton X-100, 0.25% sodium deoxycholate, 1 mM NaF, 1 mM sodium orthovanadate, 5 *μ*g/mL leupeptin, 0.2 mM phenylmethylsulfonyl fluoride, 10 *μ*g/mL aprotinin, and 0.5 mM DTT). The homogenate was centrifuged at 12000 ×g for 20 min at 4°C, and the supernatant was collected as the tissue sample lysate (TSL). The mitochondrial fraction in TSL was isolated using a Mitochondrial/Cytosol Fraction Kit according to the instruction of BioVision Inc. (SFO, CA, USA). And the protein concentration in TSL was determined using the Bradford method. The sample protein lysates were heated at 100°C for 10 min before loading and separated on precasted 7.5% SDS-PAGE. Equal amounts of sample lysates were separated by SDS-PAGE and electrophoretically transferred for 1 h onto PVDF membrane (Millipore, Billerica, MA, USA). The membrane was blocked with 5% nonfat milk in TBST buffer (20 mM Tris-HCl, pH 7.4, 150 mM NaCl, 0.1% Tween-20) and incubated 16 h at 4°C with specific primary antibodies, including anti-rat p-PI3 K, p-Akt, cytochrome *c*, Fas (CD95), caspase-3, caspase-9, caspase-12, BcL-2, BAX, *μ*-calpain, GRP 78, and CHOP (GADD153) antibodies. Subsequently, the membrane was washed with TBST buffer and incubated with the appropriate secondary antibody (HRP-conjugated goat anti-mouse or anti-rabbit immunoglobulin G). Membranes were then washed with TBST buffer and the signals were visualized using a luminescent image analyzer LAS-4000 (Fujifilm, Tokyo, Japan). *β*-Actin was used as the reference protein.

### 2.7. TUNEL Staining

A terminal deoxynucleotidyl transferase-mediated biotinylated UTP nick end labeling (TUNEL) reaction was carried out according to the protocol given by the manufacturer. Paraffin-embedded tissue sections were stained with an *In Situ* Cell Death Detection Kit (Roche Applied Science, Indianapolis, IN, USA). Briefly, paraffin-embedded sections were deparaffinized in two changes of xylene, for 5 minutes each, and then hydrated with two changes of 100% ethanol for 3 minutes each, and 95% ethanol for 1 minute each. The sections were rinsed in distilled water. For frozen sections on slides, samples were pretreated with 0.2% Triton X-100 in PBS-Tween for 30 minutes before proteinase K digestion treatment. These sections were rinsed in two changes of PBS-Tween 20 for two minutes each. The rinsed sections were preincubated in TdT reaction buffer for 10 minutes, followed by incubation in TdT reaction mixture for 1-2 hours at 37–40°C in a humidified chamber. To stop the reaction, the sections were rinsed in stop wash buffer for 10 min and then rinsed in PBS-Tween 20 for 6 min. For detection, the sections were incubated in a reaction mixture (34 mU/mL terminal transferase, 280 pmol of dATP, 90 pmole of fluorescein-11 dUTP, 30 mM Tris-HCl, 140 mM sodium cacodylate, and 1 mM CoCl_2_) (pH 7.2) for 1 h at 37°C in the dark. Cells were subsequently washed with PBS and examined under a fluorescence microscope. Positive controls were carried out by incubating sections with DNase I (3000 U/mL in 50 mM Tris-HCl, pH 7.5, and 1 mg/mL BSA) for 10 minutes at 15–25°C to induce DNA strand breaks prior to the labeling procedure. Negative controls were conducted by incubating sections with label solution only (without terminal transferase) instead of the TUNEL reaction mixture. Data were collected in triplicate and analyzed to obtain the IOA%.

### 2.8. Statistical Analysis

Data were analyzed with ANOVA and Duncan's multiple range tests using computer statistical software SAS 9.0 (SAS Institute Inc., Cary, NC, USA) to compare differences between and within groups. Different letter symbols indicate significant differences at *P* < 0.05 and the same letter symbols indicate no significant differences from each other (*n* = 3).

## 3. Results

### 3.1. The Ratio of Kidney Weight to Body Weight

**Figure 1:**
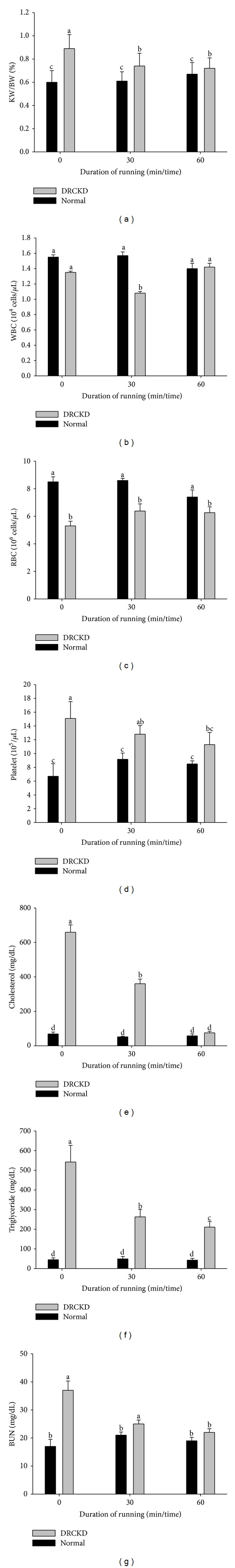
The physiological and serological parameters affected by DRCKD and exercise. (a) Ratio of KW/BW (%), (b) WBC, (c) RBC, (d) platelet, (e) cholesterol, (f) triglyceride, and (g) BUN.

DRCKD exhibited highly raised ratio of KW/BW 0.89, which was dose responsively suppressed to 0.74 and 0.72 by 30 min and 60 min exercise (EX) (Figure 1(a)).

### 3.2. Blood Cell Count

The normal control value of WBC was 1.55 × 10^4^ cells/*μ*L. Exercise did not show any effect on each control group, but in the DR-CKD groups, the 30 min EX suppressed the level to 1.08 × 10^4^ cells/*μ*L, conversely, 60 min EX restored the level to 1.42 × 10^4^ cells/*μ*L (Figure 1(b)). The RBC count was apparently reduced in the DR-CKD control to 5.3 × 10^6^ cells/*μ*L compared to 8.5 × 10^6^ cells/*μ*L of the normal control. Exercise alleviated the RBC count to 6.38 and 6.26 × 10^6^/*μ*L by the 30 min and 60 min EX, respectively (Figure 1(c)). On the contrary, the platelet count was highly raised in the DR-CKD group, which was significantly suppressed by the 30 min- and 60 min-EX to 12.8 × 10^5^/*μ*L and 11.3 × 10^5^/*μ*L, respectively (*P* < 0.05) (Figure 1(d)). 

### 3.3. The Biochemical Tests for Serum Parameters

The serum cholesterol level in the DR-CKD group was substantially raised to 659 mg/dL compared to the normal control 69 mg/dL. Exercise ameliorated the level to 360 and 75 mg/dL, respectively, by the 30 min and 60 min EX (*P* < 0.05) (Figure 1(e)). Similar to the phenomena found for the serum cholesterol, the serum triglyceride level was upregulated to 542 mg/L in the DR-CKD rats. Exercise reduced the level to 263 (by the 30 min) and 211 mg/dL (by the 60 min EX (*P* < 0.05) (Figure 1(f)). BUN was also elevated by DR-CKD, which was alleviated to 25 and 22 mg/dL by the 30 min and 60 min EX, respectively (Figure 1(g)). 

### 3.4. Bax Was Downregulated and Bcl-2 Was Upregulated

To assist the description, the overall effect of exercise on the proapototic and apoptotic parameters associated with exercise is listed in Table 1. 

**Figure 2:**
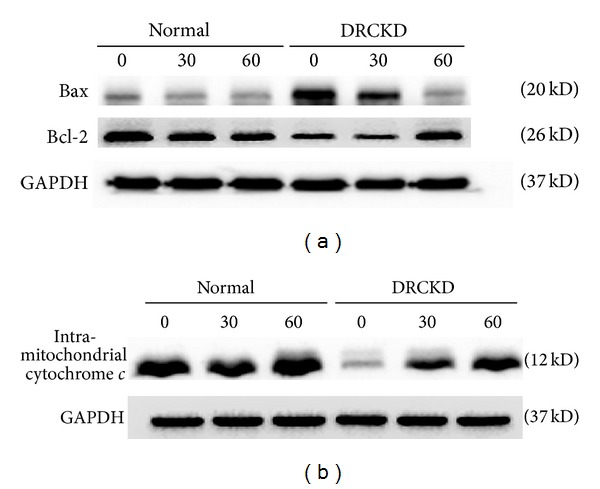
The antiapoptotic and apoptotic signals as well as the intramitochondrial cytochrome *c* levels affected by DRCKD and exercise. (a) Western blot of Bcl-2 and Bax, (b) the intramitochondrial cytochrome *c* levels.

Exercise downregulated Bax (20 kDa) and at the same time upregulated Bcl-2 (26 kDa) in DR-CKD victims (Figure 2(a)). Significant elevation of Bcl-2/Bax ratio was found in DRCKD+EX, reaching 2.9-folds and 4.1-folds for the 30 min and 60 min EX, respectively (*P* < 0.05) (Table 1). 

### 3.5. Level of Intramitochondrial Cytochrome *c* Was Effectively Alleviated by Exercise Training

The intra-mitochondrial cytochrome *c* was substantively reduced in the DRCKD control (*P* < 0.05) (Figure 2(b)). Exercise showed dose responsive beneficial effects to rescue the intramitochondrial cytochrome *c* levels (Figure 2(b)), yielding 1.8-fold and 2.00-fold increases by the 30 min and the 60 min exercise, respectively, compared to the DRCKD control (Table 1) (*P* < 0.05).

### 3.6. Cleaved Caspase-9 Expression Induced by DRCKD Was Suppressed by Exercise

**Figure 3:**
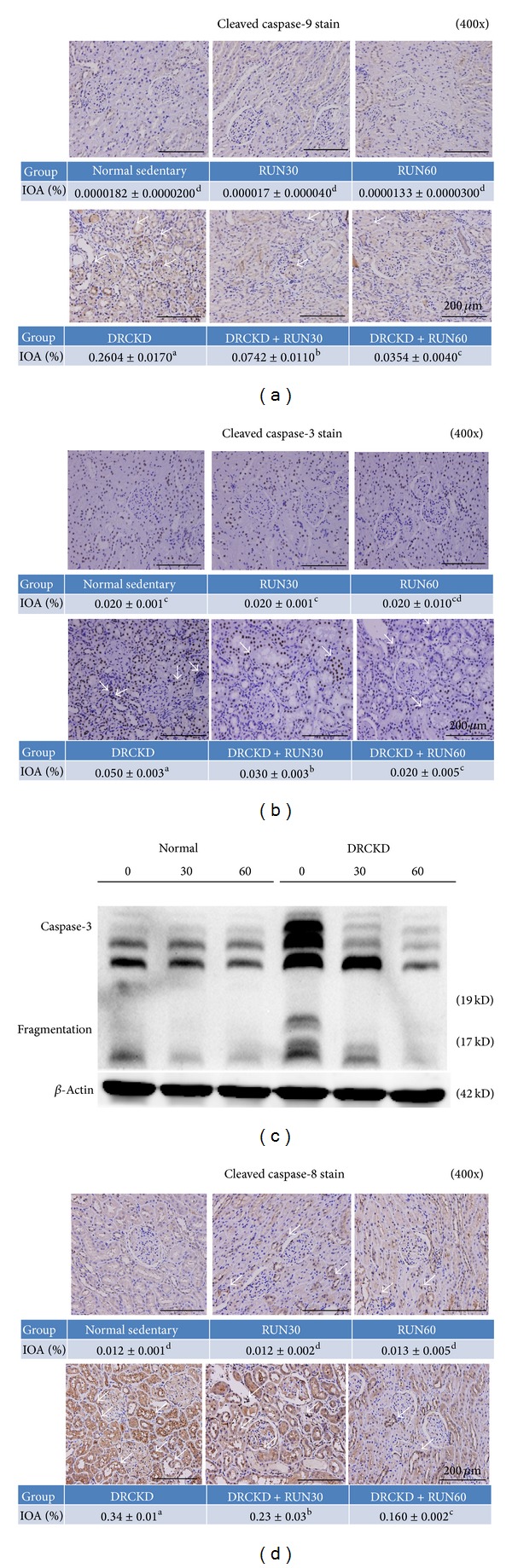
The assay of caspases. (a) IHC staining of cleaved caspase 9, (b) IHC staining of cleaved caspase 3, (c) Western blot of cleaved caspase 3, and (d) IHC staining of cleaved caspase 8 (400x). The white arrows indicate the highly upregulated expression of caspase-3, -8, and -9, which was alleviated in the DRCKD plus 60 min exercise group.

DRCKD upregulated a large amount of cleaved caspase-9 (IOA%: 0.2604 ± 0.0170) (Figure 3(a)). Exercise suppressed cleaved caspase-9 in a dose-responsive manner, yielding (IOA) 0.0742 ± 0.0110% and 0.0354 ± 0.0040% (Figure 3(a)) or folds of suppression 0.270 and 0.135, respectively, by the 30 min and 60 min exercise when compared to the DRCKD control, evidencing exercise to be rather effective for suppression of apoptosis (Table 1).

### 3.7. Cleaved Caspase-3 Induced by DRCKD Translocalized into Nuclei

DRCKD significantly translocalized cleaved caspase-3 into the nuclei of renal cells (Figure 3(b)). The translocalization of cleaved caspase-3 was ameliorated in a dose-responsive manner by exercise in the DRCKD groups. The IOA was decreased to 0.030 ± 0.003% and 0.020 ± 0.005% respectively, by the 30 min and 60 min exercise compared to the DRCKD control value 0.050 ± 0.003% (Figure 3(b)) or to 0.285- and 0.270-fold, respectively (Table 1). The fragmented caspase-3 likely involved two bands, the 19 kDa and 17 kDa fragments (Figure 3(c)). The 19 kDa identity was more exercise sensitive and disappeared soon with 30 min exercise. In contrast, the 17 kDa analogue disappeared only by 60 min exercise (Figure 3(c)) (Table 1). These findings suggest that exercise was able to effectively ameliorate renal cell apoptosis.

### 3.8. The Expression of Cleaved Caspase-8 Was Reduced in the DRCKD Exercise Groups

DRCKD significantly upregulated the cleaved caspase-8 in the sedentary DRCKD rat renal cells (Figure 3(d)). Exercise dose-responsively downregulated the expression. The cleaved caspase-8 was significantly reduced in a dose response manner in the DRCKD plus exercise groups, yielding the IOA 0.23 ± 0.03% and 0.16 ± 0.002% (Figure 3(d)), or 0.67- and 0.476 folds, by 30 min and 60 min exercise, respectively (Table 1) (*P* < 0.05).

### 3.9. DRCKD Induced ER Stress and Overexpressed Caspase-12 and *μ*-Calpain but Did Not Have Any Effect on CHOP (GADD153) and GRP78

**Figure 4:**
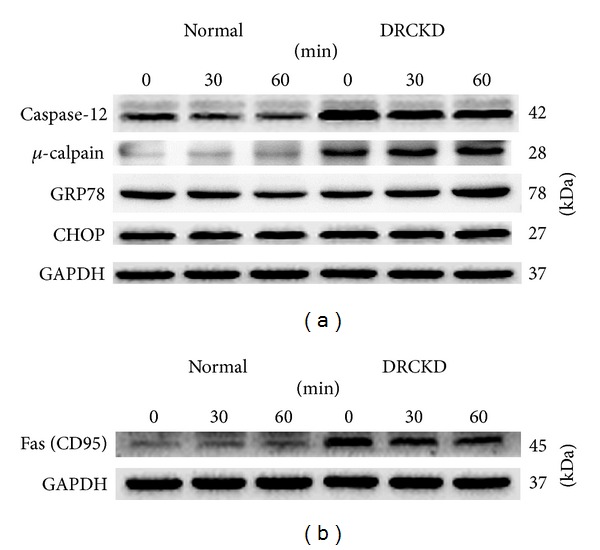
(a) Key signals expressed in ER stress and quantified data by western blotting. (b) Western blot of CD95.

Both caspase-12 (42 kDa) and *μ*-calpain were significantly upregulated in DRCKD groups (Figure 4(a)), which was significantly reduced by exercise (Table 1). However, the effect of exercise regarding these upregulations was rather limited (Table 1). On the other hand, GRP78 and CHOP (GADD153) were totally unaffected by DRCKD and/or exercise (Figure 4(a), Table 1).

### 3.10. Fas (CD95) Was Restored in the DRCKD Plus 60 min Exercise Group

Fas (CD95, 45 kDa) was significantly upregulated in renal cells of the sedentary DRCKD rats, which although it was unaffected by the 30 min exercise training it was improved slightly by the 60 min exercise (Figure 4(b), Table 1) (*P* < 0.05).

### 3.11. DRCKD Reduced the Antioxidative Capability, and Exercise Effectively Restored the Oxidative Defensive Power

DR reduced the SOD level to 7.6 ± 0.9 U/mL in the DRCKD sedentary group compared with 17.2 ± 0.6 U/mL in the normal sedentary group (*P* < 0.05) (Table 2). Exercise attenuated the SOD reduction in a dose-responsive manner such that the values were not significantly different between the control group and the DRCKD group with 60 min of exercise. Conversely, DR raised the MDA level to 9.8 ± 0.9 *μ*M in the sedentary DRCKD group, and the values for MDA in the DRCKD plus 30 min and 60 min exercise were significantly decreased from this level. However, these values were still significantly greater than in the respective control exercise groups. DRCKD reduced the antioxidative capability, and exercise was able to partially reverse the oxidation defense capacity.

### 3.12. TUNEL Staining Reveals Reduced Apoptosis in DRCKD Exercise Groups

**Figure 5:**
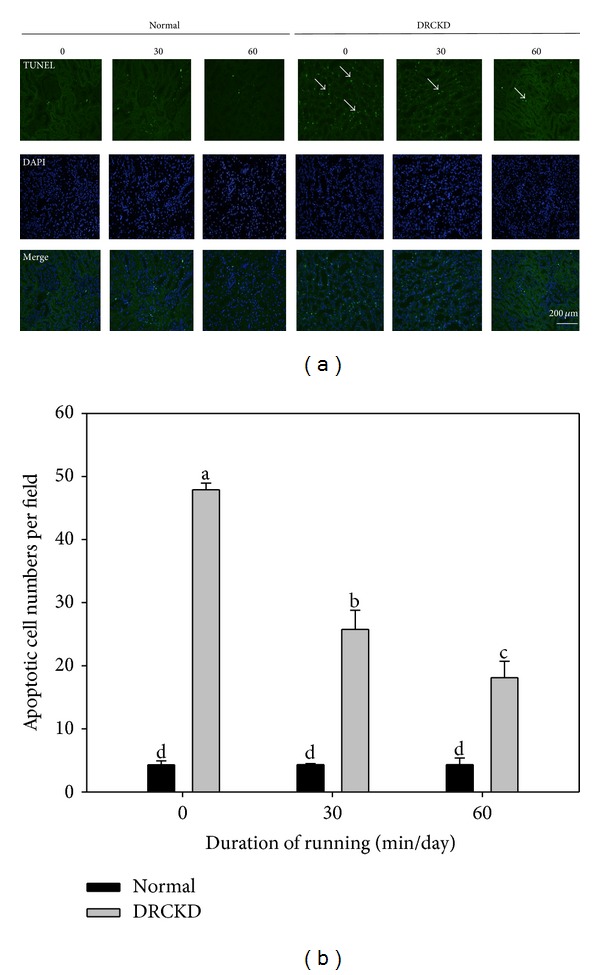
(a) TUNEL staining of DNA damage and (b) quantified apoptotic cells (400x). The white arrows indicate the apoptotic cells. The number of apoptotic cells was ameliorated in the DRCKD plus 60 min exercise group. Quantified data were collected in triplicate and analyzed with ANOVA and Duncan's multiple range tests. Different letter symbols indicate significant difference at *P* < 0.05 (*n* = 3).

DRCKD induced apoptosis (Figure 5(a)). The number of apoptotic cells reached 48 ± 4 cells/field in the sedentary DRCKD group which was reduced to 27 ± 5 cells/field and 18 ± 5 cells/field in the DRCKD plus 30 min and 60 min exercise groups, respectively, but still remained significantly greater than that in the respective control groups (Figure 5(b)).

## 4. Discussion

**Figure 6:**
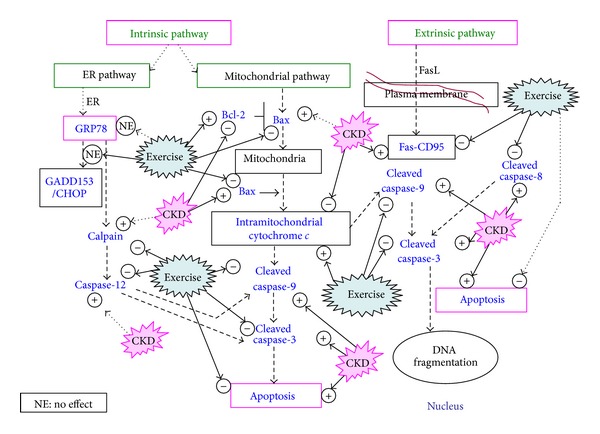
Summary of experimental results pointing to the apoptosis of CKD rat renal cells via the intrinsic and the extrinsic pathways. The verified key signals are highlighted in blue. The main apoptotic pathways are proposed via the pathways indicated by dotted bold arrows.

We showed that DRCKD+exercise elevated the Bcl-2/Bax ratio and the intramitochondrial cytochrome *c* simultaneously downregulated caspase-9, cleaved caspase-3, caspase-8, *μ*-calpain, and Fas (Table 1). Exercise reduced reactive oxygen species (ROS) (Table 2), but did not affect the ER-related CHOP and GRP78 (Table 1). For better comprehensive understanding, Figure 6. 

Recently Toyama et al. demonstrated that exercise therapy could be an effective clinical strategy to improve renal function [15]. Pechter et al. reported that exercise decreases proteinuria, cystatin c release, and ameliorated glomerular filtration rate in patients with CKD [16]. Previously, Kwak et al. [17] indicated that exercise attenuated age-induced elevation in the Bax/Bcl-2 ratio and reduced the caspase-9 level by lowering Bax protein expression while increasing Bcl-2 levels in the rat heart, which demonstrated the protective effects of endurance exercise training against increased apoptosis.

The cytochrome *c* release triggers the activation of caspase-9 in DRCKD, which was effectively suppressed by exercise trainings (Figure 6), and in the next step, caspase-3 is activated by active cleaved caspase-9 (Figure 6). A complex is formed between caspase-9 and procaspase-3, and the cleavage of procaspase-3 yields the active caspase-3 molecule [18, 19]. Cleaved caspase-3 once upregulated by DRCKD was shown effectively downregulated by DRCKD+EX (Table 1), implying the fact that exercise was potentially capable to completely ameliorate the intrinsic apoptosis occurring in renal cells with DRCKD by modulation of caspase-3. 

Present data obtained have pointed to the involvement of the mitochondrial pathway, the extrinsic pathway, and partially the ER pathway. 

### 4.1. Exercise Training Apparently Ameliorated CKD through the Mitochondrial Pathway

The mitochondrial apoptotic pathway is largely mediated through Bcl-2 family proteins, which include both proapoptotic members such as Bax, Bak, and BNIP3 that promote mitochondrial permeability, and prosurvival members such as Bcl-2 and Bcl-xL that inhibit their effects or inhibit the mitochondrial release of cytochrome *c *[20]. DRCKD simultaneously upregulated Bax and downregulated Bcl-2, suppressing intramitochondrial cytochrome *c* content, and upregulating cleaved caspase 3 and 9. Two cutting points were available in converting procaspase-3 into active cleaved caspase-3. The 19 kDa cleaved caspase-3 was found to be more sensitive to the effects of exercise than the formation of the smaller 17 kDa analogue, consistent with Fussenegger et al. [18]. 

Excessive Bax/Bak (Table 1), reactive oxygen species (ROS) (Table 2), and increased calcium ions provoked the mitochondrial cytochrome *c* release from mitochondria to cytosol. Thus, exercise appeared to be beneficial in decreasing renal cell apoptosis in part through the reduction of ROS and prevention of subsequent loss of intra-mitochondrial cytochrome *c*. 

### 4.2. The ER Stress Pathway Is Also Involved in the Recovery of DRCKD by Exercise

The ER is the site of synthesis and maturation of proteins. Various types of stress from both inside and outside cells disturb ER function, and unfolded or misfolded proteins accumulate in the ER [21]. ER stress in tubular cells affects two downstream pathways, namely, CHOP and ERK-IL6-p21. These are possible targets for suppressing progression of CKD [22]. The transcription factor CHOP/GADD153 is induced by ER stress and is involved in ER stress-induced apoptosis [21]. ER stress may initiate cell death through activation of caspases. This pathway is independent from mitochondria and death receptors and thought to be mediated by caspase-12 in ER [23]. A tubulointerstitial ER stress response, in some cases associated with tubular cell apoptosis, may occur in glomerular diseases associated with proteinuria [24]. 

*μ*-Calpain, a calcium-dependent protease, generally causes activation of human caspase-4, the counterpart of caspase-12 [25, 26]. Notably, caspase-4 triggers apoptotic pathways dependent or independent of caspase-9 and caspase-3 activation [27]. We showed that caspase-12 upregulated in DRCKD was efficiently suppressed by exercise, underlying ER stress associated with CKD [25]. Caspase-12, located on the ER membrane, has been found to induce apoptosis in response to prolonged ER stress. Caspase-12 might be activated through cleavage by a calcium-dependent protease *μ*-calpain [23]. Upon activation, caspase-12 translocates from the ER to the cytosol where it directly cleaves procaspase-9 which in turn activates the effector caspase, caspase-3 [23], therefore, bypass it the mitochondrial (cytochrome *c*, Apaf-1) apoptotic pathway [28–31]. ER stress can be provoked by a variety of pathophysiological conditions like liver cirrhosis, type 2 diabetes [21, 32], and CKD. When ER stress is overwhelming, cells undergo apoptosis. Although initially reported to be mediated by caspase-12 [33], this mechanism has been frequently challenged [34] and thus is still unclear. Herein we demonstrated that upregulated caspase-12 in DRCKD was readily suppressed by exercise training (Figure 4(a)) (Table 1). 

The upregulation of caspase-12 and *μ*-calpain induced by DRCKD was significantly but not completely improved by exercise training (Figure 4(a)) (Table 1), implying the possibility of certain crosstalk between the ER and mitochondria-dependent death pathways. 

On the other hand, inositol trisphosphate-3 (IP3) receptors may also be activated during apoptosis because it is theorized that cytochrome *c* released from mitochondria controls Ca^2+^ efflux from the ER via the IP3 receptors. This generates a positive feedback loop, whereby Ca^2+^ causes mitochondrial permeability and stimulates cytochrome *c* release [35]. 

### 4.3. The Extrinsic Pathway Was Also Modulated by Exercise to Ameliorate DRCKD

CD95, also known as Fas or APO-1, is a 45 kDa cell surface type I membrane glycoprotein belonging to a subgroup of family members that have a death domain (DD). Binding of death ligands such as Fas ligand, tumor necrosis factor (TNF) *α*, and tumor necrosis-related apoptosis-inducing ligand (TRAIL) usually induces oligomerization of the associated TNF receptors, followed by recruitment of adaptor proteins—Fas-associated death domain proteins (FADD)—to the cytoplasmic portions of the receptors [19, 36]. FADD is responsible for transmitting the death signal for apoptosis. Stimulation of CD95 results in aggregation of its DD, leading to the recruitment of FADD and caspase-8 that together with the receptor form the death-inducing signaling complex (DISC). The resulting DISC recruits multiple procaspase-8 molecules that mutually cleave and activate one another through induced proximity [34]. 

Type I cells show that the optimal formation of the CD95 DISC initiating a direct caspase cascade independent of mitochondrial changes during apoptosis. In contrast, in type II cells, DISC formation is strongly reduced [37], implicating the renal cells in DRCKD to be the type II cells. 

To conclude, exercise is beneficial to the amelioration of DRCKD, and more importantly, both the intrinsic and extrinsic pathways were involved in such a rehabilitation process. Treadmill exercises at 30 m/min for 30 or 60 min/day, 3 times per week for 11 weeks were effective in ameliorating renal cell apoptosis. The findings obtained have the potential to significantly impact the traditional management of chronic kidney disease. Specifically, exercise introduced in the early stages of renal disease may be able to substantively delay or arrest further progression of disease. Based on this, renal rehabilitation must be established as a primary component in the treatment of CKD.

## Figures and Tables

**Table 1:** The duration-dependent fold change of proapoptotic and apoptotic parameters associated with exercise.

Parameters	DRCKD + EX 30 min/DRCKD control	DRCKD + EX 60 min/DRCKD control
Bcl2/Bax ratio	2.900	4.100
Intramitochondrial cytochrome *c*	1.800	2.000
Caspase-9	0.270	0.135
Cleaved caspase-3 (19 KDa)	0.285	0.270
Cleaved caspase-3 (17 KDa)	0.710	0.417
Caspase-8	0.670	0.476
Caspase-12	0.910	0.920
*μ*-Calpain	0.800	0.757
CHOP	1.000	1.000
GRP78	1.000	1.000
Fas	1.000	0.806

**Table 2:** SOD and MDA levels in DRCKD affected by exercise*.

Duration of exercise (min)	0		30		60	
Groups	Normal	DRCKD	Normal	DRCKD	Normal	DRCKD
SOD (U/mL)	17.2 ± 0.6^a^	7.6 ± 0.9^b^	17.0 ± 0.9^a^	10.9 ± 0.8^b^	19.0 ± 0.7^a^	15.5 ± 0.9^a^
MDA (*μ*M)	2.3 ± 0.4^d^	9.8 ± 0.9^a^	2.5 ± 0.6^d^	7.0 ± 0.1^b^	2.1 ± 0.3^d^	4.8 ± 0.4^c^

*Values in the same line with different superscripts are significantly different from each other (*P* < 0.05).

## Abbreviations

BNIP3: Bcl-2/adenovirus E1B 19kD-interacting protein 3
BSA: Bovine serum albumin
C/EBPs: CCAAT/enhancer binding proteins
CHOP: C/EBP homologous protein
CKD: Chronic kidney disease
DD: Death domain
DISC: Death-inducing signaling complex
DR: Doxorubicin
DRCKD: Doxorubicin-induced CKD
EMT: Epithelial-to-mesenchymal transdifferentiation
ER: Endocytoplasmic reticulum
ERAD: ER-associated degradation
ESRD: End-stage renal disease
FADD: Fas-associated death domain proteins
FasL: Fas ligand
IOA: Integrated optical absorbance
IP3: Inositol trisphosphate 3
MDA: Malondialdehyde
PDGF-BB: Platelet derived growth factor BB
p-PDGFR: Phosphor-platelet derived growth factor receptor
PVDF: Polyvinylidene fluoride
ROS: Reactive oxygen species
SOD: Superoxide dismutase
TBARs: Thiobarbituric acid reactive substance
TGF-beta: Transforming growth factor beta
TRAIL: Tumor necrosis-related apoptosis-inducing ligand
TSL: Tissue sample lysate
TUNEL: Terminal deoxynucleotidyl transferase-mediated biotinylated UTP nick end labeling
UPR: Unfolded protein response.
